# Pure Red Cell Aplasia Associated with Thymolipoma: Complete Anaemia Resolution following Thymectomy

**DOI:** 10.1155/2018/8627145

**Published:** 2018-10-09

**Authors:** David Ferreira, Royston Ponraj, Adrian Yeung, Jillian de Malmanche

**Affiliations:** ^1^Conjoint Associate Lecturer, University of New South Wales, Sydney, Australia; ^2^Medical Department, Medical Registrar, Liverpool Hospital, Elizabeth St., Liverpool, NSW 2170, Australia; ^3^Haematology Department, Haematology Advanced Trainee, Liverpool Hospital, Elizabeth St., Liverpool, NSW 2170, Australia; ^4^Haematology Department, Haematology Staff Specialist, John Hunter Hospital, Lookout Rd., New Lambton Heights, NSW 2305, Australia

## Abstract

Pure red cell aplasia is an uncommon cause of anaemia rarely associated with thymoma. A combination of immunosuppressive therapy and thymectomy offers a potential cure. Thymectomy alone rarely results in anaemia resolution. A seventy-three-year-old male with Klinefelter syndrome presented with progressively increasing shortness of breath and anaemia. Serological testing supported primary bone marrow pathology, and a bone marrow biopsy was performed. A pure red cell aplasia was seen on bone marrow examination, and computed tomography of the chest demonstrated a thymoma. Thymectomy was performed, and histology revealed a thymolipoma. Complete anaemia resolution was achieved following thymectomy alone. This suggests that thymomas may directly mediate immune dysregulation resulting in erythroid precursor destruction.

## 1. Introduction

Pure red cell aplasia (PRCA) is a rare cytopenia characterised by a marked reduction of erythroid precursors in the bone marrow. While most cases are idiopathic, there are a number of possible secondary causes. These include lymphoid and myeloid malignancies, autoimmune disease, viral infection, drugs, and thymoma [1].

Thymomas accounts for less than ten percent of all pure red cell aplasia [1, 2]. The mechanism by which they cause PRCA is incompletely understood. Anaemia is thought to result from a paraneoplastic immune mediated destruction of erythroid precursors [2]. Thymic histological findings are variable and can include medullary thymoma, spindle thymoma, epithelial thymoma, lymphocytic thymoma, thymic carcinoma, and thymolipoma [3, 4]. Treatment involves surgical resection in combination with immunosuppressive therapy, as surgery alone is generally ineffective [3, 5]. The authors present a case of pure red cell aplasia secondary to thymolipoma with complete resolution of anaemia following surgical excision alone.

## 2. Case Report

A seventy-three-year-old gentleman presented with progressive shortness of breath over a two-month period. His medical history was significant for Klinefelter syndrome, heart failure with reduced ejection fraction, obstructive sleep apnoea, hypogonadism, haemochromatosis, and secondary polycythaemia requiring 6–12 monthly venesections. Clinical examination was unremarkable. On presentation, he had a normochromic normocytic anaemia with a haemoglobin of 82 g/L, a reticulocyte count of 2 × 10^9^/L, and an elevated haptoglobin (Table 1). Vitamin B12, folate, and thyroid-stimulating hormone studies were normal, and serum ferritin was increased (Table 2). These laboratory results, notably the markedly reduced reticulocyte count, were consistent with reduced production of red cells in the bone.

**Figure 1:**
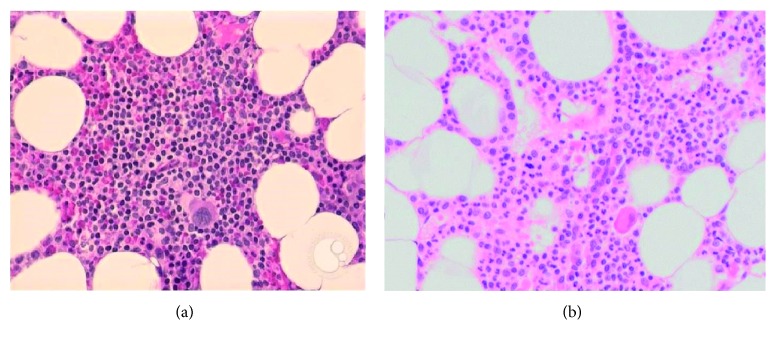
(a) Normal bone marrow biopsy demonstrating a predominance of erythroid precursors (note cells with round, dark nuclei). This image was originally published in ASH Image Bank. Peter Maslak. Normal adult bone marrow. ASH Image Bank. 2010; Trephine Biopsy-2. ©The American Society of Hematology. (b) Bone marrow biopsy taken from the patient, demonstrating marked reduction in erythroid precursors.

**Figure 2:**
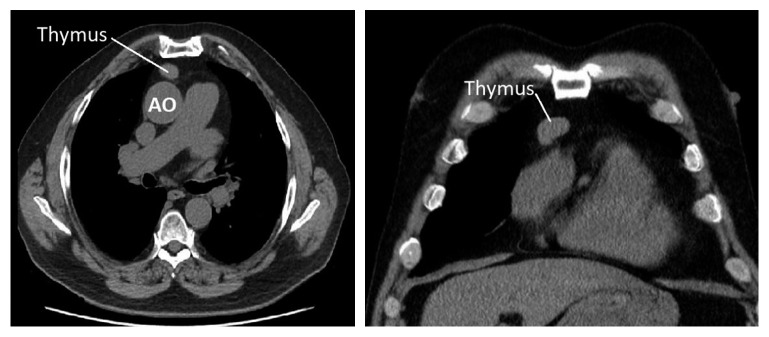
Thymoma on noncontrast chest computed tomography.

Bone marrow biopsy demonstrated a marked reduction in erythroid precursors (two percent of the differential) consistent with pure red cell aplasia (Figure 1). Normal granulopoiesis and megakaryopoiesis were evident. Autoimmune screening (ANA, ENA, dsDNA, RF, and anti-cardiolipin antibodies) and viral screening were negative (hepatitis B, hepatitis C, human immunodeficiency virus, and parvovirus B19). Serum protein electrophoresis and immunosubtraction were negative for monoclonal bands, and flow cytometry was normal. There were no recent medication changes. A chest computed tomography was performed revealing an anterior mediastinal mass consistent with thymoma (Figure 2). An elective thymectomy was arranged with a cardiothoracic surgeon, and intermittent blood transfusions were provided while awaiting surgery.

Thymectomy was performed via a median sternotomy. Histopathology demonstrated normal thymic tissue mixed with mature adipose tissue, diagnostic of thymolipoma. No inflammation, granulomata, or neoplasia was identified. Three weeks following thymectomy, the patients' haemoglobin normalised with a complete resolution of his symptoms. After a year of follow-up, the patients' haemoglobin remains normal, without immunosuppressive therapy or ongoing transfusions.

## 3. Discussion

Thymolipomas account for 2–9% of all thymic neoplasms [6]. There have been three previous reports of PRCA associated with thymolipoma [3, 7, 8]. In all three cases, patients received both surgical resection and immunosuppressive therapy prior to any improvement in haemoglobin. We present a case of pure red cell aplasia associated with thymolipoma that resolved following thymectomy alone. The mechanism behind anaemia associated with thymolipoma is not understood. Complete resolution following thymectomy suggests that thymolipomas may directly mediate erythroid precursor destruction. The underlying mechanism may be that of immune cell maturation dysregulation and subsequent autoimmune destruction [2]. Thymolipomas are associated with autoimmune diseases including myasthenia gravis, aplastic anaemia, Graves' disease, and lichen planus [9]. Moreover, patients with Klinefelter syndrome have an increased risk for autoimmune disease, associated with the XXY karyotype [10].

This case serves as a reminder that primary bone marrow pathology is differential for every patient presenting with anaemia. Simple serological screening tests provide pivotal information to guide the investigation and management of patients presenting with anaemia. While uncommon, every patient with a PRCA warrants a chest computed tomography to identify a thymoma, as thymectomy offers a potential cure.

## Figures and Tables

**Table 1:** Full blood count comparison.

	Normal range	6 months prior	Admission	3 months after thymectomy
White cells (10^9^/L)	4.0–11.0	7.5	6.4	7.0
Red cells (10^12^/L)	4.5–6.5	5.95	2.94	4.97
Haemoglobin (g/L)	130–180	160	81	150
Haematocrit (L/L)	0.38–0.52	0.498	0.243	0.466
Mean cell volume (fl)	80–100	79	83	94
Platelets (10^9^/L)	150–400	264	354	252
Neutrophils (10^9^/L)	2.0–8.0	5.2	4.4	4.6
Lymphocytes (10^9^/L)	1.0–4.0	1.2	0.9	1.4
Monocytes (10^9^/L)	0.2–1.0	1.0	0.9	0.9
Eosinophils (10^9^/L)	0–0.4	0.1	0.1	0.1

**Table 2:** Anaemia screen during hospitilisation.

	Normal range	Admission
Reticulocytes (10^9^/L)	10–100	2
Vitamin B12 (pmol/L)	130–850	228
Folate (nmol/L)	7.0–46.4	26.7
Ferritin (ug/L)	30–300	1196
Iron (umol/L)	11–30	57
Transferrin (g/L)	1.6–3.4	2.4
Transferrin saturation (%)	15–45	90
TSH^*∗*^ (mIU/L)	0.4–5.0	1.65
LDH^Ϯ^ (U/L)	120–250	236
Haptoglobin (g/L)	0.3–2.0	2.68

^*∗*^Thyroid-stimulating hormone; ^Ϯ^lactate dehydrogenase.
